# The role of copy-number variation in the reinforcement of sexual isolation between the two European subspecies of the house mouse

**DOI:** 10.1098/rstb.2019.0540

**Published:** 2020-07-13

**Authors:** Henry L. North, Pierre Caminade, Dany Severac, Khalid Belkhir, Carole M. Smadja

**Affiliations:** 1 Institut des Sciences de l'Evolution (UMR 5554 CNRS, IRD, EPHE, Université de Montpellier), Université de Montpellier, Campus Triolet, Place Eugène Bataillon, 34095 Montpellier, France; 2 MGX-Montpellier GenomiX, c/o Institut de Génomique Fonctionnelle, 141 rue de la cardonille, 34094 Montpellier Cedex 5, France

**Keywords:** reinforcement, structural rearrangements, Pool-Seq, speciation, hybrid zone, *Mus musculus*

## Abstract

Reinforcement has the potential to generate strong reproductive isolation through the evolution of barrier traits as a response to selection against maladaptive hybridization, but the genetic changes associated with this process remain largely unexplored. Building upon the increasing evidence for a role of structural variants in adaptation and speciation, we addressed the role of copy-number variation in the reinforcement of sexual isolation evidenced between the two European subspecies of the house mouse. We characterized copy-number divergence between populations of *Mus musculus musculus* that display assortative mate choice, and those that do not, using whole-genome resequencing data. Updating methods to detect deletions and tandem duplications (collectively: copy-number variants, CNVs) in Pool-Seq data, we developed an analytical pipeline dedicated to identifying genomic regions showing the expected pattern of copy-number displacement under a reinforcement scenario. This strategy allowed us to detect 1824 deletions and seven tandem duplications that showed extreme differences in frequency between behavioural classes across replicate comparisons. A subset of 480 deletions and four tandem duplications were specifically associated with the derived trait of assortative mate choice. These ‘Choosiness-associated’ CNVs occur in hundreds of genes. Consistent with our hypothesis, such genes included olfactory receptors potentially involved in the olfactory-based assortative mate choice in this system as well as one gene, *Sp110*, that is known to show patterns of differential expression between behavioural classes in an organ used in mate choice—the vomeronasal organ. These results demonstrate that fine-scale structural changes are common and highly variable within species, despite being under-studied, and may be important targets of reinforcing selection in this system and others.

This article is part of the theme issue ‘Towards the completion of speciation: the evolution of reproductive isolation beyond the first barriers’.

## 1. Introduction

### (a) Studying reinforcement at the genomic level

The evolution of reproductive isolation is necessary to initiate speciation. However, if speciation is considered complete only when gene flow is totally or almost-totally inhibited, reproductive isolation must not only evolve but reach a sufficient strength (‘complete’ reproductive isolation) [1]. Therefore, a focal aim for the study of speciation is to understand how and why reproductive isolation increases over generations. Reproductive isolation is caused by barrier traits that reduce the frequency of interspecific mating (prezygotic isolation) or reduce the fitness of hybrids (postzygotic isolation). Through reinforcement, hybrid unfitness can favour the evolution of additional isolating barriers [2]. In the strictest sense, reinforcement occurs when prezygotic barrier traits are selected in response to the fitness consequences of postzygotic barriers [3,4]. Under a broader definition, reinforcement is the enhancement of a reproductive barrier over time through the adaptive coupling of any barrier effects [2]. Regardless of the sense in which the term is used, reinforcement necessarily increases total reproductive isolation over time. Extensive empirical and theoretical work [5,6] has shown that reinforcement can and does push divergent populations ‘further along’ the speciation continuum, even leading to complete reproductive isolation under some conditions [7]. Yet the genetics of reinforcement is an emerging field. A couple of studies have identified reinforcing loci through candidate gene or QTL mapping approaches [8,9], though studies leveraging whole-genome datasets are still missing. Genomic approaches to the study of reinforcement have the potential to identify reinforcement at the genomic level that might otherwise be phenotypically cryptic, and to shed light on the identity, distribution and effect size of loci underlying reinforcing barrier traits. Ultimately, the identification of loci contributing to reinforcement can potentially help to reconstruct the history of allele divergence (standing variation in allopatry or de novo mutation in areas of hybridization) and the timing of reinforcing selection.

Recently, Garner *et al.* [10] articulated the expected genome-wide patterns of divergence and diversity under the process of reinforcement in the context of secondary contact. One prediction is that loci underlying barrier traits that contribute to reinforcement should show signatures of selection in sympatric but not in allopatric populations. A second prediction is that genetic divergence at loci underlying prezygotic reproductive isolation and targets of reinforcing selection will be elevated when allopatric and sympatric individuals of the same species are compared. This pattern of genotypic displacement corresponds to the phenotypic pattern of reproductive character displacement observed in various taxa [e.g. 11–13]—for example, the observation of positive assortative mate preference within a contact or hybrid zone, but not in allopatric populations. Critically, these expectations provide a framework for associating prezygotic barrier traits with genetic variation in the biological systems in which the phenotypic signature of reinforcement has been observed [5]. By extension, these data will shed light on the evolutionary dynamics of reinforced, and therefore strong, reproductive isolation. Given the increasing accessibility of sequence data, we are witnessing a new wave of studies that address the broader aim of identifying loci associated with barrier traits [14–16], though this effort is yet to extend to the study of reinforcement.

### (b) Copy-number variation and the evolution of reproductive isolation

When studying the genetic basis of barrier traits, the focus is still largely biased toward the identification of single-nucleotide polymorphisms (SNPs) as opposed to structural variants despite the potential of the latter to play a key role in adaptation and speciation [17–21]. In terms of research on structural variation, substantial attention has rightly been given to chromosomal inversions due to their capacity to inhibit recombination, protecting linked genes involved in reproductive isolation from inter-lineage homogenization [22–25]. However, technological limitation has long hindered the ability to associate finer-scale structural polymorphism in the genome with barrier traits [26,27], so the role of duplications and deletions (collectively, copy-number variants; CNVs) in speciation is still largely overlooked [28–30]. A number of properties make these mutations interesting candidates as a basis for barrier traits.

CNVs can contribute to the evolution of barrier traits through their effects on phenotypes and fitness. They occur at a similar rate to indels [31] despite a larger fitness effect-size [17]. Copy-number variation can contribute to phenotypic divergence by altering gene products, creating paralogues that can diverge and neofunctionalize, or by altering gene dosage [32,33]. CNVs can also act as Bateson–Dobzhansky–Muller [34–36] incompatibilities if independent pseudogeneization or neofunctionalization of duplicated paralogues in divergent lineages leads to dysfunctional genes in hybrids, [37] as observed in sympatric *Mimulus* species [38].

Additionally, CNVs may promote linkage disequilibrium among locally adapted genes, and more generally among barrier loci, by altering recombination rate. This may occur through a number of mechanisms. First, CNVs can alter chromatin structure, locally repressing recombination [39]. These CNV-induced recombination cold-spots may harbour and link alleles that reduce hybridization. Second, in the same way that chromosomal inversions promote speciation, CNVs may locally alter recombination rate simply because copy-number variation alters locus homology between homologous chromosomes and therefore the nucleotide affinity upon which recombination relies. When a tandem duplication or deletion fixes in one lineage but not another, the mutated region will no longer be homologous with that locus on the ancestral chromosome. Finally, tandem-duplicate genes are physically linked and therefore inherited as a block, so functional diversification of paralogues may be a common mechanism through which ‘supergenes’ evolve [40,41]. This echoes theoretical developments showing that genetic architectures minimizing recombination and hence promoting linkage disequilibrium between co-adapted genes, and in particular those generated by chromosomal rearrangements such as duplications, will be selected for in the context of divergence with gene flow [42–44].

The properties of CNVs mean that they should not be overlooked in genomic approaches to the study of speciation-with-gene-flow in general, and reinforcement in particular. Here we identify CNVs that may contribute to reinforced mate preferences in the hybrid zone between the two European subspecies of the house mouse (*Mus musculus*) through their expected patterns of divergence under a reinforcement scenario.

### (c) Study system

The two European subspecies of the house mouse (*Mus musculus musculus* and *M. m. domesticus*) diverged in allopatry approximately 350–500 kyr from the Indian subcontinent before coming into secondary contact in Europe approximately 5 kyr [45,46]. Prezygotic isolation is in the form of positive assortative mate preference in both sexes of both subspecies, though preference is stronger in *M. m. musculus* [12,47–50]. In both subspecies, mate preference is known to be at least partially driven by olfactory cues present in mouse urine [51,52].

Mate discrimination shows a clear pattern of character displacement both in Denmark [12] and in the broader Central European part of the hybrid zone [53]. Since postzygotic isolation has also been demonstrated in this hybrid zone, mostly in the form of reduced hybrid sterility [54–56], reproductive character displacement is in this case consistent with reinforcement. Current research efforts aim to determine the genetic basis of sexual isolation and reinforcement in the Danish part of the hybrid zone by testing expected genomic signatures of reinforcement [10]. Recent work has identified signals of selection specific to populations displaying assortative mate preference (‘Choosy’ populations from the contact zone) in genomic regions bearing receptors known to be involved in pheromone recognition (vomeronasal receptors, VRs, expressed in the vomeronasal organ) using a hitchhiking mapping approach [57]. The corresponding expected divergence between ‘Choosy’ and ‘Non-Choosy’ populations has been found at the expression level, specifically identifying a phylogenetically related group of VRs clustered on chromosome 7 [58]. Work still in progress on whole genomes confirms nucleotide divergence at those candidate VR genes but also extends candidate genomic regions to some olfactory receptor (OR) genes, expressed in the main olfactory epithelium.

To fully characterize the information impressed on the genome by the process of reinforcement, it is important to complement information obtained at the nucleotide and expression levels with measures of divergence at the structural level. This is particularly relevant in the house mouse system, in which copy-number variation has been found to be generally widespread within and between wild or wild-derived populations [39,59–63]. Moreover, olfaction, which permits mice to recognize and preferentially mate with conspecifics, is genetically encoded by large multigene receptor families including ORs and VRs [64,65]. These gene families show extensive copy-number variation in murines generally, as well as within house mouse subspecies and strains [66–70]. Copy-number divergence among house mouse Choosy and Non-Choosy populations affecting those candidate gene families and potentially other regions of the genome may therefore contribute to the evolution of choosiness in the hybrid zone.

To address this question, we leveraged the high-quality reference genome and annotation available for this species and produced whole-genome resequencing data to finely characterize copy-number variation within and between Choosy and Non-Choosy populations of *M. m. musculus* (the subspecies where the contrast in behaviour is the strongest between these two types of populations [12,48,49]). We sought to identify CNVs showing the expected pattern of divergence under a reinforcement scenario, i.e. copy-number displacement in Choosy populations, as compared to Non-Choosy populations, mirroring the observed behavioural divergence. Specifically, we aimed to: (I) test whether there exist tandem duplications or deletions that are overrepresented or fixed in Choosy populations, but underrepresented or absent in Non-Choosy populations; (II) characterize the distribution of such CNVs; (III) test whether candidate gene families involved in olfaction (OR and VR families) display the pattern of copy-number divergence consistent with reinforcement.

## 2. Material and methods

### (a) Mice, sequence data and read mapping

**Figure 1.**
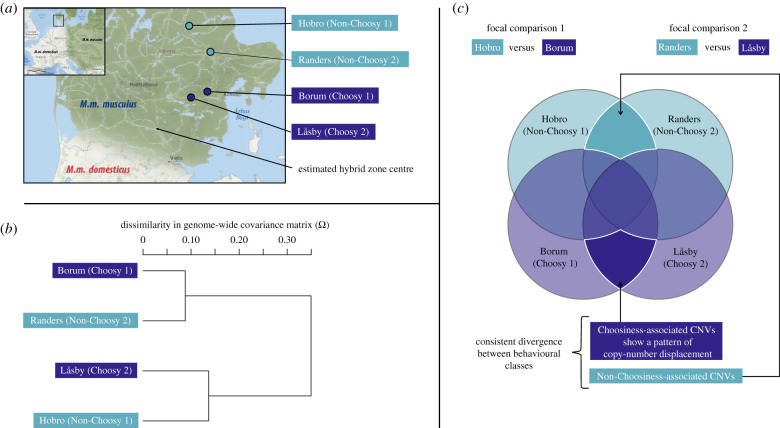
Sampling scheme (*a*) Populations of *Mus musculus musculus* sampled in localities in the *M. m. musculus - domesticus* hybrid zone (dark blue points) or further away from the centre of the hybrid zone (light blue points). The southern Choosy locality is Låsby (*n* = 27 individuals per sequenced pool), the northern is Borum (*n* = 33). The southern Non-Choosy locality is Randers (*n* = 29), the northern is Hobro (*n* = 29). (*b*) Relatedness among populations based on the genome-wide covariance matrix (*Ω*) across population allele frequencies. (*c*) Pairwise population comparisons used to identify CNVs associated with reinforcement and Venn diagram representing expectations of shared CNVs between the populations sampled. CNVs that show consistent divergence in both comparisons of Choosy and Non-Choosy populations are highlighted; they comprise two categories of CNVs: ‘Non-Choosiness-associated’ CNVs, that occur at high frequency exclusively in Non-Choosy populations, and ‘Choosiness-associated’ CNVs, our prime candidates, that occur at high frequency exclusively in Choosy populations. Choosiness-associated CNVs show a pattern of copy-number displacement and therefore show the clearest association with the evolution of positive assortative mate choice. (Online version in colour.)

Wild adult mice were trapped in Jutland, Denmark in October 2010 in several sites (indoor farms and other human dwellings) and then maintained in the laboratory under controlled conditions before being behaviourally tested and euthanized for dissection. Sampling sites represented two distinct geographical areas in Jutland, which are characterized by populations with distinct mate preference behaviours but sufficiently geographically close to avoid any geographical effect of distant allopatry: (1) the border of the hybrid zone on the *M. m. musculus* side (50 km north to the genetic centre of the hybrid zone defined in [71]) where strong assortative mating was previously documented [12,48,49] and confirmed on these newly sampled populations [50,57] (Choosy populations), (2) another area in Jutland further north from the centre of the hybrid zone, where a recent study did not find any significant directional mate preference in these sampled populations (Non-Choosy populations) [50,57]. Two populations per behavioural class were included as biological replicates, each composed of several trapping sites (four populations in total, hereafter called ‘Borum Choosy1’, ‘Låsby Choosy2’, ‘Hobro Non-Choosy1’ and ‘Randers Non-Choosy2’) (see figure 1*a* and electronic supplementary material, table S1 for a detailed sample description). For an average of 30 individuals (standard error (s.e.): 1.3 individuals) from each of the four populations, spleen was extracted rapidly after death by cervical dislocation, immersed in ethanol and stored at −80**°**C. We extracted genomic DNA from individual mouse spleens using the Macherey–Nagel kit standard protocol.

To assess genomic divergence in two independent comparisons of Choosy and Non-Choosy populations, at the level of the whole genome (genome size: 2.7 Gb), we carried out a pool-sequencing approach. Pooled samples representing each population were obtained by mixing individual DNA extracts from each population in equimolar proportions. Library preparation and sequencing were carried out at the MGX-Montpellier GenomiX platform (Institut de Génomique Fonctionnelle - Institut de Génétique Humaine, Montpellier, France). Sequencing libraries (average insert size: 478 bp (Borum Choosy1), 497 bp (Låsby Choosy2), 478 bp (Hobro Non-Choosy1) and 445 bp (Randers Non-Choosy2)) were prepared using two methods to increase sequence diversity: Illumina TruSeq DNA Sample Prep Kit v2 (three quarters of samples), and Illumina Nextera DNA sample Prep kit. Overall, three-fourths of all sequence data were obtained using the TruSeq protocol. All libraries were sequenced on an Illumina HiSeq2000 machine using a paired-end 100-bp read-length protocol, targeting an average raw coverage per pool of 30X. *Bowtie2* v2.2.3 [72] was used to map reads to the mouse reference genome assembly GRCm38 [73] and *SAMtools* [74] was used to format binary sequence alignment/map (.bam) files and remove PCR-duplicate reads. We used the program *BayPass*, which provides an accurate estimation of the genome-wide covariance matrix (Ω) across population allele frequencies, to infer the genome-wide population structure of our dataset [75] (figure 1*b*).

### (b) Identification of differentiated copy-number variants from pooled paired-end sequencing data

We detected CNVs significantly and consistently differentiated in allele frequency between Choosy and Non-Choosy populations using a combination of read depth, read-pair orientation and insert size information, adapting the approach developed by Schrider *et al.* [76,77] to detect copy-number variation from pooled data. This approach (detailed in electronic supplementary materials, figures S1–S3) is limited to the identification of multinucleotide deletions and tandem duplications. Briefly, within each of the four pools, tandem duplications were identified as clusters of ‘everted’ inserts, because read-pairs spanning a tandem duplication become everted when mapped to the reference genome (electronic supplementary material, figure S1). By leveraging the fact that read-pairs spanning a deletion appear unusually distant when mapped to the reference genome, deletions were identified as clusters of adjacent, disproportionately large inserts [78] (electronic supplementary material, figure S2). All CNVs identified were supported by a stringent minimum of five discordant read-pairs. Two proxies for allele frequency were used for those mutations: the number of read-pairs supporting each CNV event and local read depth of coverage (excluding highly repetitive regions). The chromosomal coordinates of these mutations could then be compared between populations to report the presence or the absence of each mutation as well as the difference in allele frequency. Two CNVs were considered comparable between two populations if their coordinates differed by less than half of their mean size.

#### (i) Identification of significantly divergent CNVs between pairs of populations

CNVs that significantly differed in frequency between a pair of populations were defined using two criteria: (1) the 5th percentile of the difference in the number of supporting read-pairs and (2) the 5th percentile of deviation from a 1 : 1 ratio of local read depth. For the latter criterion, the significance cut-off was determined empirically by randomly selecting genomic regions of a similar length to the CNV in question and measuring the ratio of read depths, selecting the top and bottom 5% cut-offs from the resulting distribution. The set of lengths used were 50 bp, 100 bp, 150 bp, 200 bp, 0.5 kb, 1 kb, 2 kb, 5 kb and 8 kb. CNVs with both a significant difference in supporting read-pairs and a concordant difference in read depth ratio were considered as significantly differentiated between a pair of populations.

#### (ii) Identification of CNVs showing consistent divergence between Choosy and Non-Choosy populations

To identify CNVs consistently differentiated between Choosy and Non-Choosy populations within the focal subspecies *M. m. musculus*, beyond population-specific differences, we only retained CNVs that show consistent, ‘parallel’ divergence in the two independent pairwise comparisons involving Choosy populations (Borum-Choosy1 versus Hobro-Non-Choosy1; Låsby-Choosy2 versus Randers-Non-Choosy2; ‘consistent divergence’ in figure 1*c*). Specifically, we disregarded all CNVs showing either insignificant allele frequency differences in at least one comparison, or significant allele frequency differences in both replicate comparisons but not in the ‘same direction’ in each comparison. Disregarded CNVs that show allele frequency divergence in opposing ‘directions’ include, for example, a deletion that showed a significantly higher frequency in Borum-Choosy1 in the first comparison but showed a significantly higher frequency in Randers-Non-Choosy2 in the second comparison. We refer to the two replicate comparisons of Choosy and Non-Choosy populations as the Focal Test. To ensure that the CNVs matched across replicate comparisons were comparable, we required that they overlap over most of their genomic range. We retained and classified two different categories CNVs according to their degree of overlap, to separate mutations that are strictly identical in their coordinates from those that slightly differ in their coordinates. CNVs assigned to first category (identical by state) had matching start and end chromosomal coordinates in both comparisons of Choosy and Non-Choosy populations. CNVs assigned to the latter category (non-identical by state) occurred at partially overlapping loci in both comparisons. Specifically, for CNVs classed as non-identical by state, the observed CNV size in each comparison differed by less than 5% of the mean CNV size across the two comparisons. All unmatched CNVs (those for which CNV sizes differed by more than 5% in the two comparisons) were removed and disregarded.

In addition to the Focal Test (comparing sets of Choosy and Non-Choosy populations), we performed a Control Test in order to determine whether there was an enrichment of parallel divergent CNVs in the Focal Test compared to population comparisons with no *a priori* expectations regarding reinforcement. The Control Test also consists of two population pairwise comparisons, but this time within behavioural classes (Hobro-Non-Choosy1 versus Randers-Non-Choosy2; Borum-Choosy1 versus Låsby-Choosy2). Since there is no *a priori* expectation for non-neutral evolutionary forces to cause consistent divergence between these pairs of populations, the Control Test is therefore a null expectation for the number of loci that diverge repeatedly across replicated population comparisons. We also used the Control Test to remove false positives from the Focal Test: CNVs that we find to be consistently divergent in the Focal Test but also found to consistently divergent within behavioural classes in the Control Test were removed from the final list of candidate CNVs.

#### (iii) Choosiness-associated CNVs as prime candidates

CNVs showing consistent divergence between Choosy and Non-Choosy populations in the Focal Test correspond to CNVs either associated with Choosiness (‘Choosiness-associated’ CNVs) or associated with Non-Choosiness (‘Non-Choosiness-associated’ CNVs; figure 1*c*). Choosiness-associated CNVs are those either fixed or at high frequency in both Choosy populations. Non-Choosiness-associated CNVs are those either fixed or at high frequency in both Non-Choosy populations. The presence or absence of these CNVs in these populations was defined in relation to the reference genome (see electronic supplementary material, figures S1 and S2). The house mouse reference genome, produced from the classical C57BL/6 J inbred strain, contains alleles that can be considered as a representative of *M. m. domesticus* from non-hybrid zone populations [79–82]. As a consequence, it can be considered as representing the ancestral state under a reinforcement scenario. Under this assumption, variants sharing the same structural allele between the reference genome and *M. m. musculus* Non-Choosy populations (ancestral state) and showing a different allele in *M. m. musculus* Choosy populations (derived state) show the clearest association with the evolution of mate choice. This is because their pattern of divergence matches the pattern of copy-number displacement that is expected to be produced by reinforcement. Specifically, copy-number displacement will occur when reinforcing selection acts on barrier traits such as mate preference exclusively in sympatric populations to increase the frequency of CNVs that contribute to those traits (either de novo mutations or CNVs pre-existing in allopatric populations). In summary, Choosiness-associated CNVs show the pattern of copy-number displacement expected under reinforcement and are therefore of primary biological interest.

### (c) Genomic distribution and characteristics of differentiated CNVs

On Choosiness-associated CNVs (identical and non-identical by state), we first performed a permutation test (accounting for ‘masked regions’ of low coverage that were excluded in upstream analyses) to assess whether mutations were distributed heterogeneously, i.e. whether the observed number of CNVs per 10 kb was larger or smaller than expected by chance. We also performed a permutation test of the duplications and deletions to test for enrichment in genes annotated in the UCSC mm10 known gene database while accounting for masked regions of the genome [83–85]. Finally, in order to test whether Choosiness-associated CNVs were present in regions of reduced or enhanced recombination, we performed permutation tests to measure over-representation in ‘cold-spots’ of recombination reported by Morgan *et al.* [39] and in recombination ‘hot-spots’ reported by Smagulova [86]. To perform these permutation tests, we used *BEDtools* [87], the *RegioneR* [83] package in R version 3.5.1 [88] and custom *bash* scripts. The R package *ChromPlot* was used to visualize the genomic distribution of CNVs, [89] and *Gviz* was used to visualize gene models of Ensembl transcripts [90].

### (d) Functional enrichment analyses

The Ensembl *Variant Effect Predictor* [91] was used to determine the predicted effect of Choosiness-associated CNVs (e.g. coding sequence variants) and to identify gene annotations (Ensembl and RefSeq IDs) in their vicinity (within 1 kb upstream and downstream of each gene). This allowed us to explicitly test for the presence of genes that belong to candidate multigene families (ORs and VRs) and to test for overlap of divergent CNVs with genes expressed in the *M. m. musculus* vomeronasal organ that show differential expression between behavioural classes [58]. We used the program *DAVID* [92] to perform functional enrichment analysis for gene IDs nested within one or several annotation terms. In *DAVID*, annotation terms are derived from 14 public annotation sources (e.g. gene ontology terms, sequence general features, bio-pathways). Significantly enriched annotation terms were those supported by a *p*-value < 0.01 after adjustment for multiple testing using the Benjamini–Hochberg procedure implemented in *DAVID*.

## 3. Results

### (a) Copy-number variation between populations and behavioural classes of *M. m. musculus*

Our first aim was to test whether there exist tandem duplications or deletions that are overrepresented or fixed in Choosy populations, but underrepresented or absent in Non-Choosy populations. To that end, we first identified CNVs within populations. Second, we retained the subset showing significant allele frequency differences between populations. Finally, among that subset, we retained CNVs showing consistent divergence across replicate comparisons of Choosy and Non-Choosy populations, and in particular those overrepresented or fixed in Choosy populations—i.e. CNVs showing the expected pattern of copy-number displacement (figure 1*c*).

#### (i) Deletion and tandem duplication frequencies within populations

Distant read-clusters (indicative of deletions) were widespread but variable in frequency in all four populations (mean: 177 078 clusters/population, s.e.: 42 060; electronic supplementary material, figure S4). Everted read-clusters (tandem duplications) were much rarer in all populations (mean: 7718 clusters/population, s.e.: 1389). Across all populations, a total of 708 310 deletions and 30 870 tandem duplications were observed. The Hobro sample contained the most tandem duplications (11 459) and deletions (265 828) while Randers contained the fewest tandem duplications (4960) and the fewest deletions (75 989).

#### (ii) Copy-number variant frequency differences between populations

We identified CNVs that significantly diverged in frequency in the two independent comparisons of Choosy and Non-Choosy populations of *M. m. musculus* (electronic supplementary material, figures S5 and S6). In the first comparison (Borum Choosy1 versus Hobro Non-Choosy1), 273 tandem duplications and 49 590 deletions differed significantly in frequency, reflecting the average difference in frequency between duplications and deletions in all populations at step 2 (see electronic supplementary material). When Låsby Choosy2 and Randers Non-Choosy2 were compared, a similar number of tandem duplications were significantly divergent in frequency (176), though fewer divergent deletions were observed (38 927). Numbers of the same magnitude were found in the Control Test: thousands of divergent deletions (54 620 in the Choosy versus Choosy comparison; 56 084 in the Non-Choosy versus Non-Choosy comparison) and hundreds of divergent tandem duplications (418 and 119 tandem duplications in the two comparisons of the Control Test, respectively; electronic supplementary material, figure S5).

#### (iii) Copy-number variants that show consistent divergence between Choosy and Non-Choosy populations

To identify the subset showing consistent divergence between behavioural classes, CNVs were matched across comparisons. Matched CNVs were either identical by state in both comparisons, or non-identical by state (note that for CNVs in the latter class, the observed CNV size in each comparison differed by less than 5% of the mean CNV size across the two comparisons). Of 49 590 deletions that diverged between Borum and Hobro (first Choosy versus Non-Choosy comparison in the Focal Test), 2378 matching deletions were also divergent in the same **‘**direction**’** with respect to behavioural class in the Randers–Låsby comparison (second Choosy versus Non-Choosy comparison in the Focal Test) (electronic supplementary material, figure S6 and table S2). These parallel divergent deletions are not specifically enriched compared to the background level of population divergence (*p* > 0.05, one-tailed binomial test with expected success rate of 0.05). Of this set of 2378 parallel divergent deletions in the Focal Test, 554 overlapped with deletions divergent in the Control Test (electronic supplementary material, table S3); after removing these false positives, we could confirm 1824 deletions showing consistent divergence between Choosy and Non-Choosy populations (electronic supplementary material, figure S6 and table S4). Of 1824, 367 had coordinates identical by state across replicate comparisons; the rest were non-identical by state in the Focal Test (electronic supplementary material, table S4). A similar proportion (approx. 18%) of divergent deletions were identical by state in the Control Test (electronic supplementary material, table S3). Notably, the vast majority of divergent deletions (1668 of 1824) were fixed in both Choosy populations and entirely absent in both Non-Choosy populations (electronic supplementary material, table S4).

Of the 273 tandem duplications that diverged between the first pair of Choosy and Non-Choosy populations (Borum versus Hobro), just seven were also divergent in the same direction with respect to behavioural class in the replicated comparison (Randers versus Låsby) (electronic supplementary material, figure S6 and table S4). There was no evidence of enrichment of parallel divergent tandem duplications (one-tailed binomial test, *p* > 0.05). None of these seven tandem duplications were strictly identical by state across replicate comparisons, instead showing a consistent pattern of convergent evolution. In the Control Test, similar counts were observed: four divergent duplications were observed, none of which identical by state. There was no overlap in the Control and Focal Tests of tandem duplications; thus all seven tandem duplications identified in the Focal Test were retained as showing consistent divergence between Choosy and Non-Choosy populations. All divergent tandem duplications in both the Control and Focal Test showed parallel fixed differences (electronic supplementary material, table S4).

#### (iv) Copy-number variants associated with the behavioural shift toward assortative mating

Of the 1824 deletions that showed consistent Choosy–Non-Choosy divergence, 480 were associated specifically with Choosiness (dark blue in figure 1*c*). Of these 480, 440 were non-identical by state. The majority of Choosiness-associated deletions (453 of 480) were fixed in Choosy populations and entirely absent in Non-Choosy populations. Of the seven tandem duplications that showed consistent differentiation (all non-identical by state), four were Choosiness-associated, all of which fixed in Choosy populations and absent in both Non-Choosy populations. Choosiness-associated CNVs are represented in electronic supplementary material, figure S6 and listed in electronic supplementary material, table S5.

### (b) Size and genomic distribution of copy-number variants associated with Choosiness

**Figure 2.**
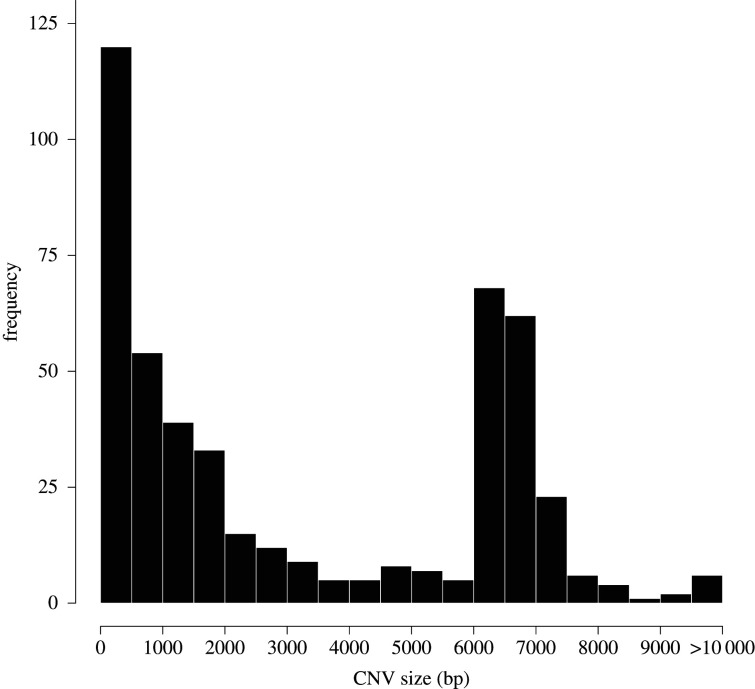
Size distribution of Choosiness-associated tandem duplications and deletions. Of 484 Choosiness-associated CNVs, 480 were deletions (mean size: 3308.8 bp, s.e.: 135.19 bp) and four were tandem duplications, ranging from 315 bp to 2487 bp (mean: 870.25 bp, s.e.: 540.90 bp).

Our second aim was to characterize the distribution of divergent deletions and tandem duplications. There was substantial variation in the size of the 484 Choosiness-associated CNVs (mean: 3309 bp, s.e.: 135 bp; figure 2). It should be noted that this distribution does not include all duplications (e.g. interchromosomal transpositions, as observed by Ishikawa *et al.* [93]), but only those that occur in tandem.

**Figure 3.**
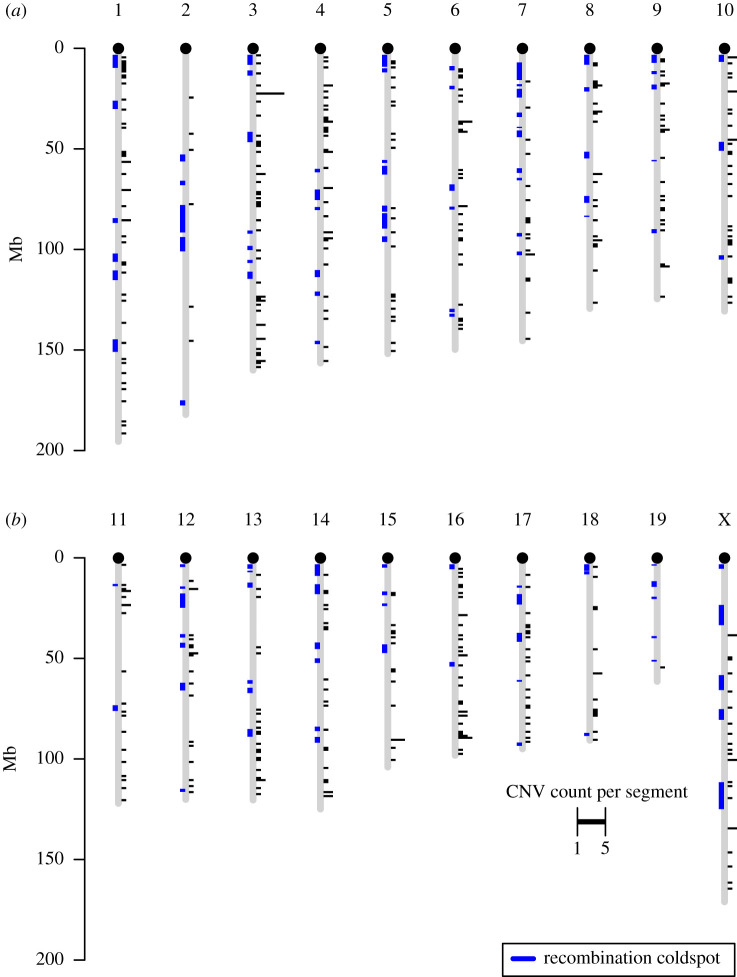
Genomic distribution of Choosiness-associated tandem duplications and deletions. Black bar height (right) indicates the count of CNVs within each 10 kb genomic segment (minimum 1, maximum 5). Blue regions (left) indicate recombination cold-spots observed in the Diversity Outbred hybrid mouse population by Morgan *et al.* [39]. (Online version in colour.)

These Choosiness-associated CNVs were distributed throughout the genome, occurring on all chromosomes analysed (figure 3). Using the expected number of deletions per megabase assuming a random genome-wide distribution, a permutation test showed that Choosiness-associated CNVs were not more clustered than expected by chance (*p* = 0.7978, 10 000 permutations). Of 484, 57 divergent CNVs occurred in a recombination cold spot and none occurred in a recombination hotspot—neither of these co-occurrences were greater than expected by chance (*p* *=* 0.6525 and *p* = 0.1873, respectively, each with 10 000 permutations; figure 3 and electronic supplementary material, figure S7).

### (c) Several Choosiness-associated CNVs occur in candidate gene families

We observed 480 Choosiness-associated deletions and four Choosiness-associated tandem duplications. A permutation test showed that Choosiness-associated deletions were significantly under-enriched in genic regions (198 occurred in genic regions; *p* < 0.05, *z* = −2.453, 10 000 permutations) (electronic supplementary material, table S6). Applying the available *Ensembl* gene IDs for deletions and tandem duplications combined in the *DAVID* software, 10 annotation terms showed significant enrichment (Benjamini–Hochberg adjusted *p* < 0.01; electronic supplementary material, table S7). The enriched sets of genes are associated with synapse function, plasma membrane function, phosphoprotein production, alternative splicing and protein localization to the cell surface.

**Figure 4.**
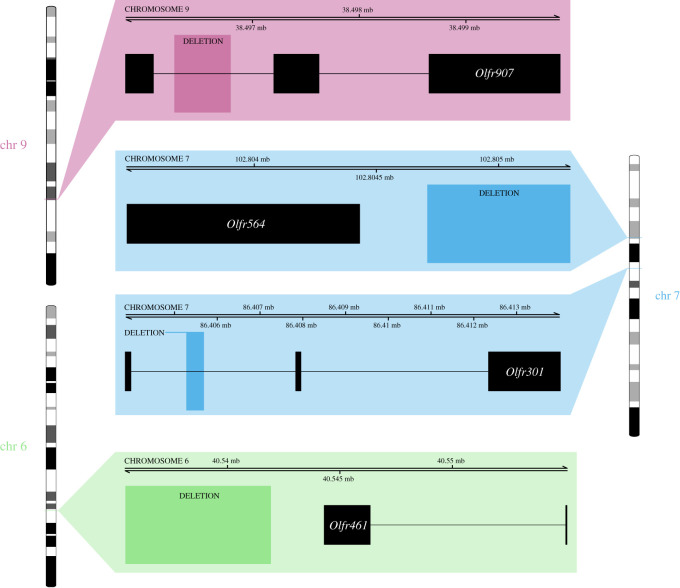
Choosiness-associated deletions within 1 kb of olfactory receptor genes. Labelled regions indicate the coordinates at which deletions associated with choosiness occurred. Black boxes indicate the chromosomal coordinates of exons, and lines indicate introns, for the four olfactory receptor genes that occurred within 1 kb of these deletions associated with Choosiness (the Ensembl transcripts displayed here as black boxes are *Olfr301-201*, *Olfr907-203*, *Olfr461-203* and *Olfr564-201*). The deletion in *Olfr907* and those near *Olfr461* and *Olfr564* were fixed in both Choosy populations and absent in both Non-Choosy populations. The deletion in *Olfr301* showed extreme frequency differences in both comparisons, though was not completely fixed/absent in the second comparison of the Focal Test. (Online version in colour.)

Our third aim was to test whether CNVs occurred in candidate gene families involved in olfaction (in particular, ORs and VRs). No association was found with VRs, but four of the 484 Choosiness-associated CNVs occurred within 1 kb of a OR gene, all of which were deletions and all of which were classed as non-identical by state, meaning that these Choosiness-associated deletions occurred at overlapping but slightly different coordinates in each replicate comparison of the Focal Test. The first was an approximately 412 bp deletion in the intronic region of *Olfr301* on chromosome 7, the second was an approximately 527 bp deletion in the intronic region of *Olfr907* on chromosome 9 (figure 4 and electronic supplementary material, table S6). The other two deletions occurred within 1kb of OR genes: an approximately 4469 bp deletion was within approximately 240 bp of *Olfr461* on chromosome 6, and an approximately 584 bp deletion occurred within approximately 279 bp of *Olfr564* on chromosome 7 (figure 4 and electronic supplementary material, table S6). Three of four OR deletions were fixed in both Choosy populations and absent in both Non-Choosy populations, with the exception of the deletion in *Olfr301*, which showed a fixed difference in the first comparison of the Focal Test (Hobro versus Borum) but a quantitative, significant allele frequency difference between populations in the second comparison (Randers versus Låsby). Since ORs are mostly expressed in the main olfactory epithelium, it is not surprising that none of these candidate OR genes showed differential expression in the vomeronasal organ when Choosy and Non-Choosy populations were compared by Loire *et al.* [58]. However, two tandem duplications (approx. 300 bp in size) occurred in the intronic region of *Sp110*, which did show differential expression in the study by Loire *et al.* [58]. These duplications (both non-identical by state) were proximate on chromosome 1, separated by approximately 1 kb.

Considering only the subset of 40 deletions that were both Choosiness-associated and classed as identical by state, approximately one third occurred in genes compared to the proportion observed across all divergent deletions. The Choosiness-associated deletions classed as identical by state and occurring in genic regions (electronic supplementary material, table S6) were mostly protein coding, however none were candidate OR and VR genes.

## 4. Discussion

### (a) Extensive copy-number variation within and between populations

In the study of speciation, the most basic hope for the genomics era is to better associate the evolution of adaptive traits, and in particular barrier traits, with underlying shifts in genetic diversity [94]. SNPs are an important metric of genetic diversity and have come to represent a standard form of data in population genomics. Variation in copy number, however, is rarely analysed despite the fact that it has been found to be widespread among natural populations [62,95] and despite the fact that sequencing data used to identify SNPs carry information about copy-number variation [78]. We used Pool-Seq data to assess copy-number variation among populations and associated this variation with the evolution of barrier traits through reinforcement.

Tens to hundreds of thousands of CNVs were identified in each of the four populations sampled (each with a mean individual sample size of 30, electronic supplementary material, table S1). In a study of European populations of *M. m. domesticus*, Pezer *et al.* sampled eight individuals per population and identified approximately 4000–10 000 CNVs within populations using a sequencing depth and PCR-validation approach [59]. By contrast, Morgan *et al.* [39] observed 1749 CNVs in a sample of 6886 mice from the Diversity Outbred mouse stock using a read-depth approach different to ours, whereas Locke *et al.* [62] observed 9634 autosomal CNVs in the genotyping array data of a more genetically diverse dataset of 290 individuals—both laboratory and wild-derived strains. Fundamental differences in the methodology and scope of CNV detection, the number of individuals sampled, and the effective population size make it difficult to compare our results to other studies. Perhaps due to the fact that this Pool-Seq method assesses CNVs genome-wide, and because we sampled many individuals per pool, we appear to observe a relatively high frequency of CNVs within wild populations using read-pair orientation in addition to read depth (step 2 of our pipeline, see electronic supplementary materials).

Between populations, tens of thousands of CNVs diverged in frequency. Using a method based on the same principle for detecting CNVs, Schrider *et al.* [77] identified hundreds of CNVs that differed between pairs of populations of *Drosophila melanogaster* and *D. serrata*, separately. Given that the mouse genome is approximately 20-fold larger than that of *Drosophila*, the CNV frequency we observe is still fivefold larger on a CNV/bp basis despite a more conservative approach taken to identify CNVs here in terms of the minimum number of supporting inserts per CNV (see the electronic supplementary material). Again, however differences in effective population size and sample size mean that this is an indirect comparison.

The baseline frequency of deletions greatly exceeded that of tandem duplications. The number of tandem duplications that showed consistent divergence between Choosy and Non-Choosy populations and an association with choosiness were proportionally also less frequent by roughly one order of magnitude. This result was surprising, given that both deletions and tandem duplications are caused by the same process of aberrant recombination. Dopman & Hartl [96] observed an approximately 2 : 1 bias of deletions to duplications in a study of *Drosophila melanogaster*, attributing this to polymorphic deletion bias or, possibly in addition, a methodological bias in their detection method (a microarray comparative genome hybridization approach). Schrider *et al.* also observed a approximately 2 : 1 bias of deletions to tandem duplications using a similar method to ours [77]. Although we introduce a clustering algorithm that groups duplications and deletions in a more similar manner to that applied by Schrider *et al.* (see electronic supplementary material figure S3), our approach, too, may be biased toward the detection of deletions. While deletions are detected as clusters of read-pairs that are significantly far apart (as defined by the upper first percentile of the distribution of insert sizes in the sequencing pool), tandem duplications are identified as everted read-pairs. This means that, at the first step, an arbitrary threshold of significance is applied when identifying deletions, whereas tandem duplications are identified qualitatively. Although we have ensured that our pipeline analyses deletions and duplications in as similar manner as possible, any differences in the probability of identifying these read-pair clusters at the first step will inevitably alter the efficiency with which deletions or tandem duplications are observed. For example, when applying a read-mapping algorithm, penalties must not be applied to everted reads if this method is to be used (in our case they were not) or else tandem duplications will not be detectable. The maximum fragment length for valid paired-end alignment (in our case 600 bp, parameter*-X* in *Bowtie2*) must not be too large, or else deletions (distant read-pairs) will go undetected. Further exploration of the potential biases of this method could quantify the effect of mapping parameters by varying them. Nonetheless, our results ultimately confirm previous findings of the ubiquity of copy-number variation in wild mouse populations [59,60,62] and provide additional support for the idea that CNVs occur at a frequency that warrants their analysis as potential targets of selection [31,60,77].

### (b) A pattern of copy-number displacement is observed in the hybrid zone

Our first aim was to test whether there exist tandem duplications or deletions showing the expected pattern of divergence under a reinforcement scenario, as set out by Garner *et al.* [10]. Using two biological replicates per behavioural class allowed us to first identify CNVs showing consistent and significant divergence between Choosy and Non-Choosy populations, beyond population-specific patterns of differentiation. Within these consistently divergent CNVs, we focus on Choosiness-associated CNVs, which show the expected pattern of copy-number displacement (figure 1*c*). As for any allele, inter-population variation in genome-wide frequency is most likely explained by variation in effective population size and interpopulation connectivity. Because we made multiple comparisons between populations, interpopulation variation in genetic diversity only makes our estimate of the proportion of reinforcement-associated CNVs more conservative. This Focal Test is also conservative as the representative populations being compared were more related to one another than were the two behavioural classes (figure 1*b*). Using a Control Test (comparisons within behavioural classes), we were able to remove false positives showing parallel divergence unrelated to the behavioural contrast, narrowing down candidate CNVs to those only specific to the Choosy–Non-Choosy comparison and ultimately identifying the prime candidates uniquely associated with the derived trait of choosiness.

The vast majority of Choosiness-associated deletions, and all Choosiness-associated divergent tandem duplications, were fixed in one behavioural class and absent in the other. This frequency of fixed differences (greater than 90% for deletions, 100% for tandem duplications) was replicated when populations were compared within behavioural classes in the Control Test, demonstrating that a pattern of population structure is captured by copy-number variation. By contrast, Pezer *et al.* [59] found that a smaller proportion (approx. 10%) of detected CNVs were population-specific in a study of European *M. m. domesticus*. However, due to the nature of our approach, arbitrary thresholds for statistical significance in the difference of allele frequencies between populations will influence the ratio of fixed versus quantitatively divergent CNVs (see electronic supplementary materials, Step 5). The fact that most Choosiness-associated CNVs showed qualitative differences between behavioural classes, however, means that they make strong candidates as alleles associated with the evolution of mate choice.

Another striking feature of Choosiness-associated CNVs was the high frequency of variants that were categorized as ‘non-identical by state’, i.e. those with strong but not strict overlap in their coordinates across replicate comparisons of Choosy and Non-Choosy populations. By contrast, less than 10% of Choosiness-associated CNVs that were identical by state (i.e. those with strictly identical coordinates; electronic supplementary material, table S5). CNVs identical by state show a pattern of divergence consistent with a differentiation in allele frequency from shared standing variation, whereas CNVs non-identical by state show a pattern consistent with convergent evolution. Therefore, our results imply that the majority of Choosiness-associated CNVs may have arisen either through convergent copy-number differentiation or through the mutational size-expansion or size-contraction of CNVs, followed by the differentiation of CNV sizes between populations. Both explanations imply a high mutation rate. Convergent evolution at CNV ‘hotspots’ has been described in several detailed case studies, [97,98] often attributed to variation in the rate of aberrant recombination [99]. While we did not find an overall effect of recombination rate variation when explaining the genome-wide distribution of CNVs, it is nonetheless possible that fine-scale recombination hotspots could lead to recurrent deletions or tandem duplications, or that the recombination rate data available (measured in laboratory strains) does not reflect the recombination landscape of the wild populations we measured. Other mechanisms of local recurrent mutation, such as DNA fragility, may also contribute [100].

It must be noted, however, that the method used here cannot perfectly estimate frequency of CNVs identical by state. Because read-pair clusters are grouped into putative CNV events in each of the two comparisons of the Focal Test independently (see electronic supplementary materials, Step 2), local variance in read depth may slightly lengthen or shorten the apparent size of the putative CNV event. So, because read depth can vary stochastically, a hypothetical Choosiness-associated deletion in Choosy population 1 may appear to be shorter than a homologous deletion in Choosy population 2 because a distant read-pair in the putative CNV region was unobserved by chance in population 1. This implies that the frequency of CNVs identical by state reported here is a lower estimate; the frequency of convergent CNVs should be interpreted with this in mind. Further ‘noise’ in the precise identification of CNV coordinates may be added by the matching of overlapping but non-identical-by-state-CNVs between populations within a comparison (see electronic supplementary materials, Step 3). However, since most CNVs of interest are fixed in one behavioural class and absent in the other (and therefore fixed in one population and absent in the other, in both comparisons), they do not need to be ‘matched’, so this secondary source of error is in this case minimal.

### (c) Processes that potentially explain copy-number displacement in the hybrid zone

Barrier traits are often observed to be stronger in populations that co-occur with heterospecific populations—a pattern termed reproductive character displacement [4]. It follows that genetic variation contributing to barrier traits will mirror this pattern of divergence (genotypic displacement) [10]. Through reinforcement, selection against unfit hybrids will lead to the differentiation of phenotypes that promote assortative mating (and underlying genotypes) in hybrid zones relative to an ancestral state observed in allopatry. However, while the pattern of character displacement can reflect a process of reinforcement, reinforcement is not the only possible cause of character displacement and phenomena unrelated to reinforcement or speciation generally can cause patterns of character displacement [101]. Howard [4,101] set out criteria to distinguish among processes leading to reproductive character displacement, which have largely been met in this system [12,50]. Setting such criteria is more challenging when investigating patterns of genotypic displacement if the link between genotype and barrier trait is unknown, because both directional and stochastic processes cause differentiation in allele frequency between contact and allopatric populations.

Several lines of evidence suggest that genetic drift explains most of copy-number variation among the populations we sampled. First, only a small proportion (2378 of 49 590) of deletions that differed between the first Choosy–Non-Choosy comparison also diverged in frequency in the second comparison of the Focal Test. Since an excess of parallel divergent deletions compared to the background level of divergence among populations was not observed, we can reject selection as the dominant force driving allele frequency differences [e.g. 76]. Second, the average numbers of significantly differentiated CNVs in the Focal Test were not qualitatively different from what was observed in the Control Test (see electronic supplementary material, figure S5). Since the Control Test provides a baseline expectation of neutral divergence in CNVs, selection cannot explain the number of CNV showing allelic divergence in the Focal Test. Thus, although assessing the respective impact of selection and drift on copy-number divergence is beyond the scope of this study, it is plausible that genetic drift explains most copy-number variation among the studied populations. However, these results do not exclude the potential for reinforcing selection to drive frequency differences between behavioural classes for some CNVs. Given that (i) there is established evidence of selection against hybridization in this hybrid zone, [54–56] (ii) behavioural classes do not share more genetic variation than the populations compared (figure 1*b*), and (iii) the expected pattern of copy-number displacement was observed in candidate OR genes (figure 4), the CNVs identified here clearly represent loci of interest at which future analyses could formally test for natural selection as a consequence of reinforcement.

Whether other forms of divergent selection could have driven copy-number displacement at some loci between Choosy and Non-Choosy populations is another important question. Indeed, any unmeasured form of spatially varying selection may have influenced genetic differentiation between behavioural classes. By using replicates of populations that are known to show distinct mating preference differences despite being closely related and inhabiting a similar environment, we minimize the risk of confounding effects. However, because the Non-Choosy populations sampled further away from the centre of the hybrid zone also necessarily occur at higher latitudes, genetic divergence between Choosy and Non-Choosy populations could be associated with latitudinal adaptation, as opposed to reinforcement alone. Mack *et al.* [102] have demonstrated latitudinal adaptation in the closely related *M. m. domesticus* at a scale of hundreds to thousands of kilometres in North America, corresponding to mean annual temperature differences between 4°C and 21°C. Here, no sampled population is separated by more than approximately 50 km, differing in mean annual temperature by approximately 1°C. Therefore, to the extent that selection explains part of copy-number divergence between Choosy and Non-Choosy populations, we expect latitudinal adaptation to play an insubstantial role relative to the effect of reinforcing selection at this geographical scale.

### (d) Frequency and distribution of Choosiness-associated copy-number variants

As discussed above, far fewer Choosiness-associated tandem duplications were observed compared with deletions, because the baseline count of tandem duplications within pools was significantly lower (see electronic supplementary material, figure S4). While both tandem duplications and deletions can inhibit or alter the expression levels of genes, only duplications can lead to the expansion of gene families through the neofunctionalization of paralogues. So, based on the results we present, the evolution of assortative mate choice is not explained by the expansion of gene families through tandem duplication in hybrid zone populations. This does not rule out the possibilities of gene family expansion through other modes of duplication, such as translocation. We also observed that Choosiness-associated CNVs were less likely to occur in genic regions than expected by chance. Therefore, to the extent that CNVs alter genes, the relatively high frequency of deletions we observe, and their genomic distribution, suggests that CNVs mostly alter pre-existing biological functions through shifts in expression levels, occasionally inhibiting functions through genic truncation.

Choosiness-associated CNVs occurred on all chromosomes and were not more clustered than expected by chance. The mutational process causing copy-number variation implies that variation in recombination rate may be an important factor in explaining their genomic distribution, [39] though we found that variation in recombination rate could not explain the distribution of Choosiness-associated tandem duplications and deletions. It should be noted, however, that recombination rate data were not available for the populations we sampled; the genetic distance between wild populations and the strains in which recombination rate is known may obscure a real relationship between CNV density and recombination rate.

### (e) Possible functional consequences of Choosiness-associated copy-number variants

Our final aim was to test whether candidate gene families involved in olfaction (OR and VR families) display the pattern of copy-number displacement consistent with reinforcement. Previous findings obtained through a comprehensive investigation of gene expression in the vomeronasal organs of the same Choosy and Non-Choosy populations had identified expression divergence at some VRs associated with Choosiness in the house mouse hybrid zone [58]. Although OR expression in the main olfactory epithelium is yet to be studied, it is also possible that ORs are involved in the evolution of choosiness in the hybrid zone. These two gene families being known to be subject to repertoire size variation in *Mus musculus* [66–70], we tested whether expression divergence affecting some pheromone receptors in the hybrid zone could originate from copy-number variation.

No Choosiness-associated CNV occurred in VR genes, indicating that the CNVs we identified cannot explain expression differentiation at these loci between the different behavioural classes [95], nor can it explain VR gene family expansion or diversification. VR expression differences between Choosy and Non-Choosy *M. m. musculus* populations is therefore likely due to regulatory rather than structural change. However, two independent tandem duplications were observed in the intronic region of *Sp110*, which, like some VRs, shows differential expression in the vomeronasal organ between Choosy and Non-Choosy populations [58]. Those duplications may therefore explain the change in gene expression at this gene. Acting in the regulation of gene transcription [103], this gene could potentially influence regulatory changes underlying the evolution of assortative mate choice.

Consistent with our hypothesis, some olfactory receptors showed copy-number divergence between Choosy and Non-Choosy populations. There was no enrichment of this class of genes (electronic supplementary material, table S7), but four Choosiness-associated CNVs (all deletions, all with coordinates non-identical by state in the two independent comparisons of the Focal Test) occurred in or near four OR genes. Two occurred within a few hundred bp of *Olfr461* and *Olfr564*, and two occurred in intronic regions of *Olfr907* and *Olfr301*. If these CNVs had a functional impact, it would alter expression levels but not the protein coding sequence. We predict that these OR genes show differential expression between Choosy and Non-Choosy *M. m. musculus* individuals in the Danish hybrid zone. Confirmatory analyses of RNA concentration in tissues including the main olfactory epithelium would test this prediction. If OR expression variation does play an important role in mate recognition and choice in this system, these CNVs represent strong candidates as targets of reinforcing selection.

In addition to candidate genes, Choosiness-associated CNVs occurred in or near hundreds of genes (electronic supplementary material, table S6) that represent potential future candidate mutations affecting mate choice in this system. In the set of all Choosiness-associated CNVs, there was enrichment for genes associated with synapse function and alternative splicing, both of which conceivably affecting mate choice via behavioural variation or gene expression regulation (electronic supplementary material, table S7). There was also enrichment for genes associated with synapse function and alternative splicing, both of which conceivably affecting mate choice via behavioural variation or gene expression regulation. Among the set of Choosiness-associated CNVs with coordinates identical by state in both replicate comparisons, 19 deletions occurred in genic regions (electronic supplementary material, table S6). These included truncations of the genes *Slc6a16*, *Grid2* and *Kcnn2*, which all play a role in neurotransmission and could conceivably alter behavioural phenotypes [104–106].

We do not seek to make claims about the biological mechanism of the CNVs associated with mate preference, however we do point to various CNVs that are clearly interesting candidates for formal tests of selection to investigate. With functional validation, these variants may be linked to phenotypes of interest. By investigating the role of copy-number variation, we have revealed otherwise cryptic genetic variation associated with assortative mate preference and demonstrated that this variation alone is not sufficient to explain previously reported differences in gene expression associated with positive assortative mate preference [58].

### (f) Genomics and the role of structural variation in the study of reinforcement

Conceptual and technological advances hold the promise of scanning the genome for the footprint of reinforcing selection in a range of systems, thereby enhancing our understanding of how barriers to reproduction arise, interact and increase in strength [10]. However, a reductive conception of the genome as a fixed, linear series of loci at which SNPs arise will limit the scope of empirical investigation. Copy-number variation represents a substantial yet under-studied component of genetic variation, with a strong potential to contribute to the evolution of barrier traits and the strengthening of reproductive isolation. We have shown that information capturing copy-number variation can be extracted from pooled sequencing data, and that these data can be used to characterize extensive and otherwise cryptic genetic variation associated with reinforcing selection. Approaches similar to this can be used on pre-existing and future datasets to better understand the heritable basis of traits that increase reproductive isolation.

## Supplementary Material

Supplementary material

## Supplementary Material

Sample description

## Supplementary Material

Table S2 : Consistently divergent CNVs in the Focal Test

## Supplementary Material

Table S3 : Consistently divergent CNVs in the Control Test

## Supplementary Material

Table S4 : Final set of consistently divergent CNVs between Choosy and Non-Choosy populations

## Supplementary Material

Table S5 : Choosiness-associated CNVs

## Supplementary Material

Table S6 : Choosiness-associated CNVs that occur within 1 kb of a gene

## Supplementary Material

Table S7 : Significantly enriched annotation terms of genes in which Choosiness-associated CNVs occurred

## Data accessibility

Raw mapping files (BAM files) and metadata have been uploaded to the Short Read Archive (SRA, NCBI) under the bioproject ID PRJNA603262. The scripts used in this study are available as a Git repository available at https://gitlab.mbb.univ-montp2.fr/khalid/poolcnvcomp.
