# Supplementary material for: The role of copy-number variation in the reinforcement of sexual isolation between the two European subspecies of the house mouse
Source: Philos Trans R Soc Lond B Biol Sci. 2020 Jul 13;375(1806):20190540. doi: 10.1098/rstb.2019.0540 (PMC7423270; doi:10.1098/rstb.2019.0540)
Supplement: Supplementary material [file rstb20190540supp1.pdf]

The role of copy-number variation in the reinforcement of sexual isolation between the two European subspecies of the house mouse. North *et al.* (2020) Philos. Trans. R. Soc. Lond., B, Biol. Sci.

Supplementary material

Pipeline summary

The pipeline we apply here is described by Schrider *et al* (2013, 2016), available at: https://github.com/andrewkern/poolDiffCNV

We apply the Python scripts by Schrider *et al.* with some alterations, with the aim of clustering and analysing tandem duplications and deletions (collectively, Copy-Number Variants, CNVs) in a similar manner such that their frequencies are more directly comparable. These scripts are available on our Git repository: https://gitlab.mbb.univ-montp2.fr/khalid/poolcnvcomp

We alter the Python scripts for Step 1 to read *.bam* files. We replace Step 2 scripts with a new clustering algorithm. The *distanceCutoff* parameter is altered in Step 3 and Step 4 is altered to read .bam files. The pipeline is otherwise the same. Bash scripts, available on our GitLab repository, were used in downstream analyses.

Another notable difference between the pipeline used by Schrider *et al* and our analysis is that we perform two “tests”, each consisting of two comparisons. In the “Focal Test” we conduct two comparisons of Choosy and Non-Choosy populations. In the “Control Test” we conduct two comparisons within behavioural classes (Choosy 1 vs Choosy 2; Non-Choosy 1 vs Non-Choosy 2).

The Basic idea of CNV detection is as follows:

Illumina sequencing produces read-pairs: two nucleotide sequences (each 100-bp long in our case), one read in the 5’→3’direction and the other read 5’←3’. That is, the read-pair has an inverted orientation. If a tandem duplication occurs, a read-pair that spans the end of the first copy and the beginning of the second will not be mapped to the reference in the correct orientation; the read-pair orientation is *everted* (5’←3’ and 5’→3’; Supplementary Figure S1). Therefore, clusters of everted read-pairs can be used to identify tandem duplications and read-depth information can be used to validate duplication events (Cooper, Zerr, Kidd, Eichler, & Nickerson, 2008; Guan & Sung, 2016; Schrider et al., 2013). If a deletion occurs in a given pool relative to the reference genome, the insert size will be greater than expected when mapped to a reference genome (Supplementary Figure S2). Just as with duplications, there will be a significant difference in read depth at the putative deletion locus between the pool and a pool in which the deletion is absent. Note that we use the terms “duplication” and “deletion” only in relation to the reference genome assembly *GRCm38*, because copy number variation is detected through the comparison of pooled DNA sequences to the reference. “Discordant” refers to everted or distant read pairs collectively.

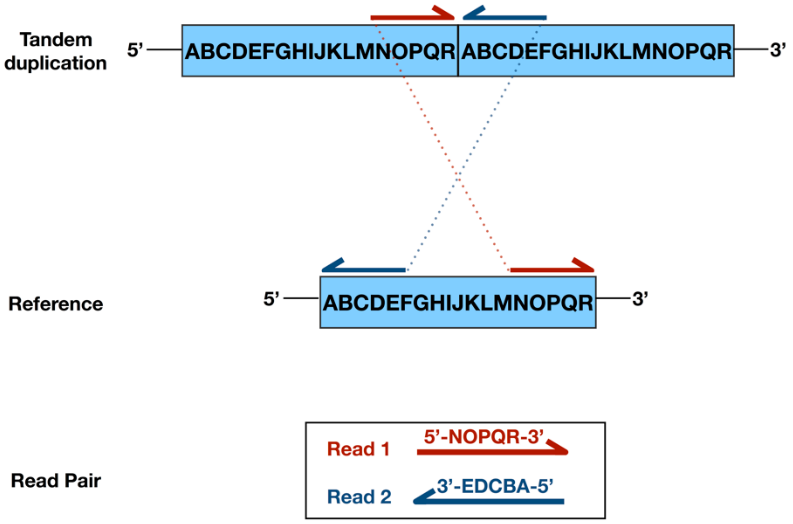

Supplementary Figure S1
Letters represent a sequence of pseudo-nucleotides. Reads 1 and 2 belong to the same read-pair (*i.e.* insert), which typically map in an inverted orientation. A read-pair spanning a duplicated region will map to the reference in an everted orientation.

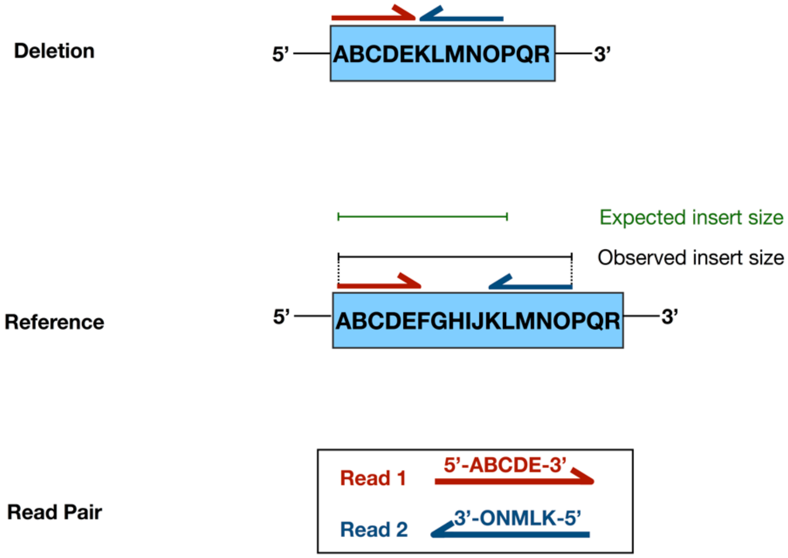

Supplementary Figure S2
A read-pair spanning a deleted region will have an extreme insert size relative to other read-pairs when mapped to a reference. These figures are adapted from Figure 3a by Cooper *et al.* (2008).

Step 1: Identify distant and everted read-pairs

We first identify distant reads. For each pool and for each of 20 chromosomes (19 autosomes and X; insufficient coverage could be achieved for Y), we subset inserts that exceeded the parameter *insertSizeCutoff*, which represents the upper first percentile of the insert size distribution for each respective pool. For Step 1d we apply Schrider's *findEvertedInserts.py*

Similarly, Step1 for everted reads (Step 1e) was applied using the *findEvertedInserts.py* script from Schrider *et al* (2013), altered to read .bam files.

Step 2: Cluster distant and everted read-pairs within pools to identify deletions and tandem duplications

Multiple discordant read-pairs caused by a CNV event will map to the same location, so nearby discordant reads need to be clustered to distinguish distinct CNV events from singleton discordant read-pairs. Rather than using the .py script developed by Schrider *et al.* (2013), we implemented a similar but faster approach (Supplementary Figure S3).

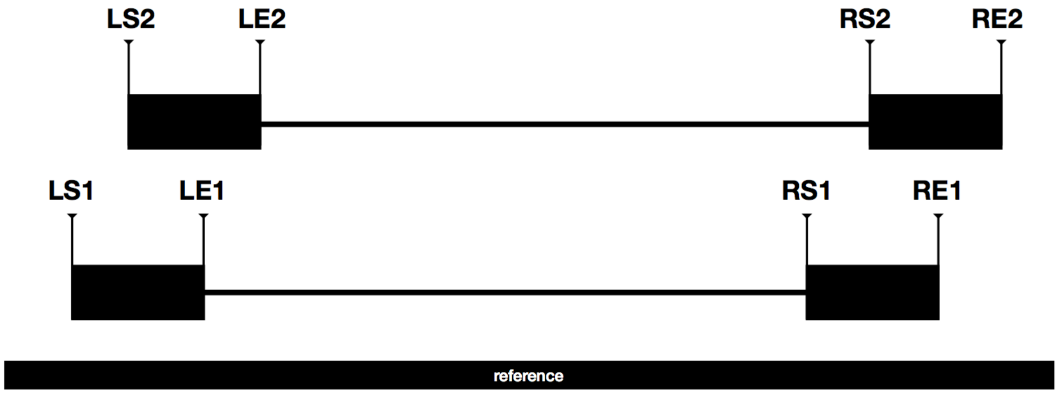

Supplementary Figure S3
A cartoon of two read-pairs (inserts), labelled 1 and 2, caused by the same deletion event. Each insert consists of a left (L) and a right (R) read, with known start (S) and end (E) coordinates. The insert size is the distance from LS to RS. Adjacent distant reads of similar size indicate the same deletion event. Therefore, as in Schrider *et al.* (2013), two adjacent read-pairs assigned to the same deletion event must not differ in size more than the parameter *insertSizeDiffCutoff*. Where SD indicates standard deviation, this parameter is 2[SD(insert size)] for a given pool. Unlike Schrider *et al.* (2013), we then simply apply the rule LS2 < RS1 for any read-pairs belonging to the same cluster, given that inserts are sorted by start position. This is because the deletion must occur between LE1 and RE1. We also require that deletions size is between 50 bp and 10 kb, and that each deletion is supported by at least 5 read-pairs.

Step 3: Match corresponding CNVs between pools and calcualte the nornalised number of supporting read-pairs in each pool

The first criterion to identify differentiated CNVs between two populations is the difference between the number of read-pairs supporting a mutation in each population, used as proxy for allele frequency difference. In order to compare CNV events across pools, clusters must be matched to corresponding CNV events across pools. Clusters are considered part of the same event if their coordinates are within a distance, *distanceCutoff*, from one another when two pools are compared. We set the *distanceCutoff* parameter to equal half the mean of the two CNVs being compared. Apart from redefining *distanceCutoff*, we use the script written by Schrider *et al.* (2013; '*combineDistantClustersAccrossPools.py*'). This step therefore reports whether each polymorphism present in one sample is present in the other, and also reports the number of read-pairs supporting the event in each population. The number of supporting read-pairs is normalised by the empirical read depth in each pool to account for inter-pool differences in depth.

Step 4: Calculate relative read depth differences

The second criterion to identify deletions that differ in frequency between two populations is the read-depth difference between two populations. In Step 4, for each deletion event and duplication event we calculate the read depth for each of the populations being compared. The ratio of these depths is then calculated for a given population comparison. For this calculation we ignore masked regions of the genome that are highly repetitive. We apply a variation of *countReadPairsInCNV.py* altered such that it can read .bam files using PySam. The masked regions .bed file was accessed via: https://genome.ucsc.edu/cgi-bin/hgTables

Step 4.5: Append additional allele frequency information to each duplication and deletion event

In order to know whether the read depth ratio is significantly different between pools, we compared observed read depth ratios to an empirical distribution. The empirical distribution was generated by measuring read depth ratios for many regions that belong to a specific size class. Each observed CNV event was assigned to a size class. Each size class has a known upper 95% threshold and lower 5% threshold for the expected difference in read depth, based on empirical sampling of the data. CNVs that exceed the upper or lower thresholds are labelled.

Confirmed deletions and duplications are those for which the read depth ratio is extreme in the same "direction" as the difference in the number of supporting inserts. For example, if there is a duplication in pool A, there should be more everted read pairs in A compared to B and a higher read depth in A compared to B. CNVs that do not conform to this are labelled as "false positives."

Step 5: Label CNVs that are divergent between Choosy and Non-Choosy populations

In the 5th step we subset the number of putatively differentiated deletions and tandem duplications between two focal populations depending on the two criteria described above: extreme read depth ratios and extreme differences in the number of supporting inserts.

Step 6: Subset CNVs that are highly differentiated across replicate comparisons.

Based on the values assigned in Steps 4 and 5, we subset the positions of deletions that are highly differentiated between Choosy and Non-Choosy populations **and** which have overlapping positions. The same principle is applied to the Control Test comparisons.

For each test, a distinction is made between coordinates that overlap perfectly ("identical by state"; those which have identical start and end chromosomal coordinates), and those that overlap almost-perfectly ("non-identical by state"; those which overlap and differ by no more than 95% of their mean size).

The bedtools closest -d command is used to determine the extent of overlap between a given CNV in the first pool and the closest CNV in the second pool. The ratio of the two CNV sizes is then calcualted. If this value is between 0.95 and 1.05, the CNV is retained. If, additionally, the difference in both the start and end coordinates of the two comparisons is also zero, these CNV are marked as "identical by state."

Removal of CNVs that occur in both Control and Focal Tests at the end of Step 6

For the group of Focal CNVs that are >95%-similar in size (i.e. including those that are "identical by state"), those that intersect with the same class of CNVs produced by Step 6 in the Control test are removed to produce the final set of consistently divergent CNVs across replicate pairwise comparisons of Choosy and Non-Choosy populations (see Supplementary Tables).

Supplementary figures

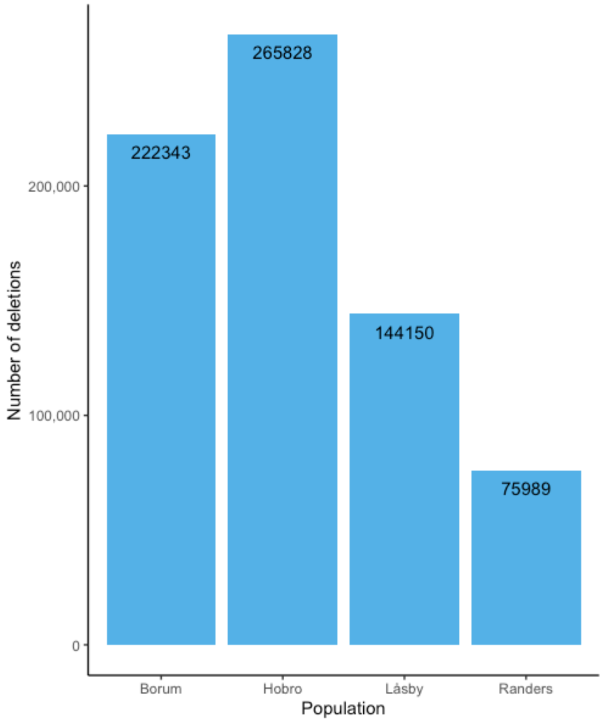

Supplementary Figure S4a Number of deletions observed in each population at the completion of step 2.

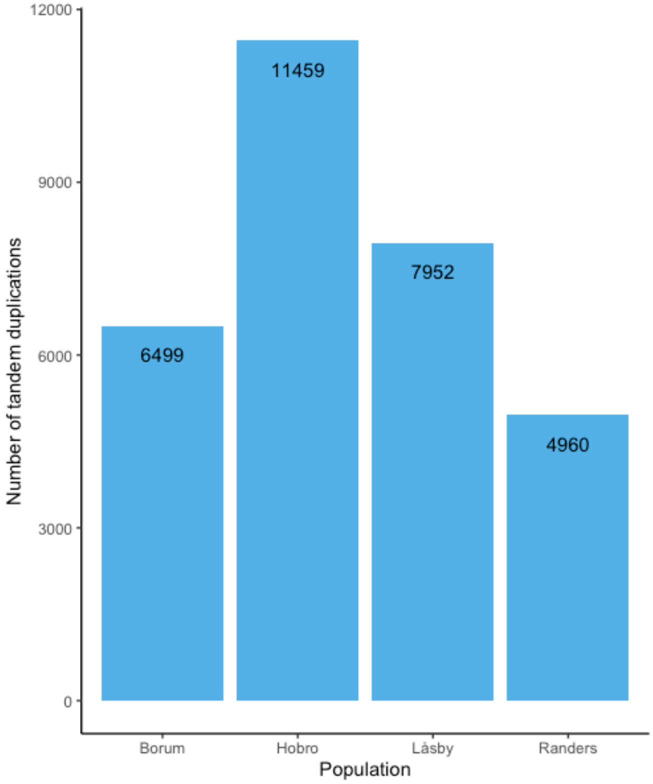

Supplementary Figure S4b Number of tandem duplications observed in each population at the completion of step 2.

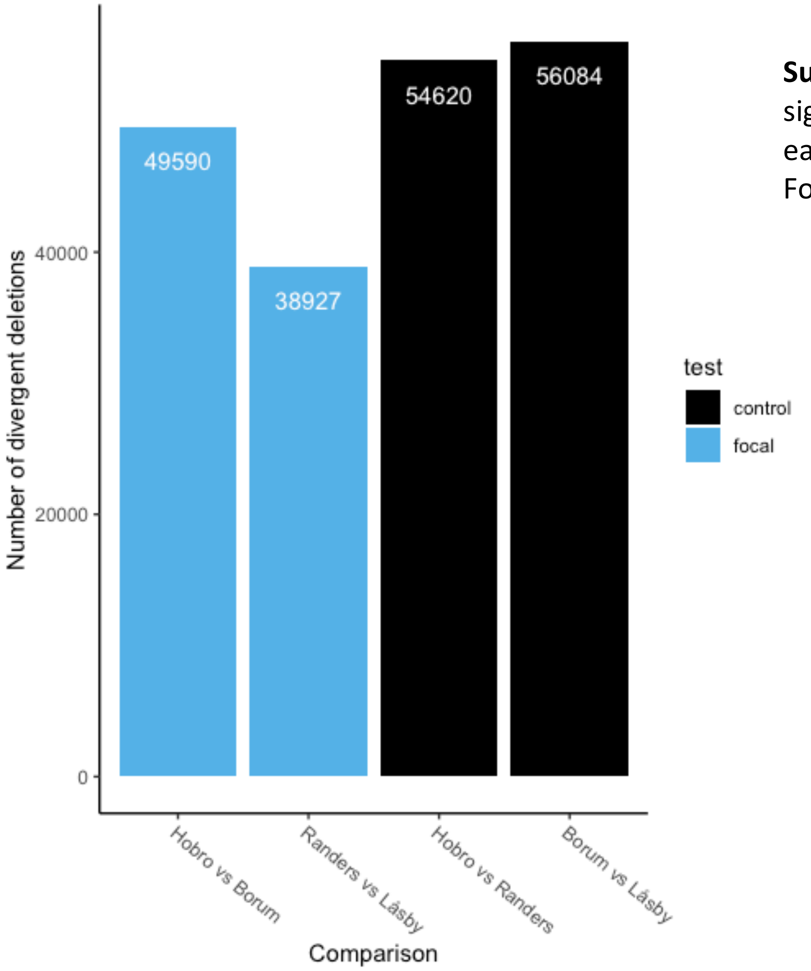

Supplementary Figure S5a Number of significantly divergent deletions between each pairwise population comparison of the Focal Test and of the Control Test.

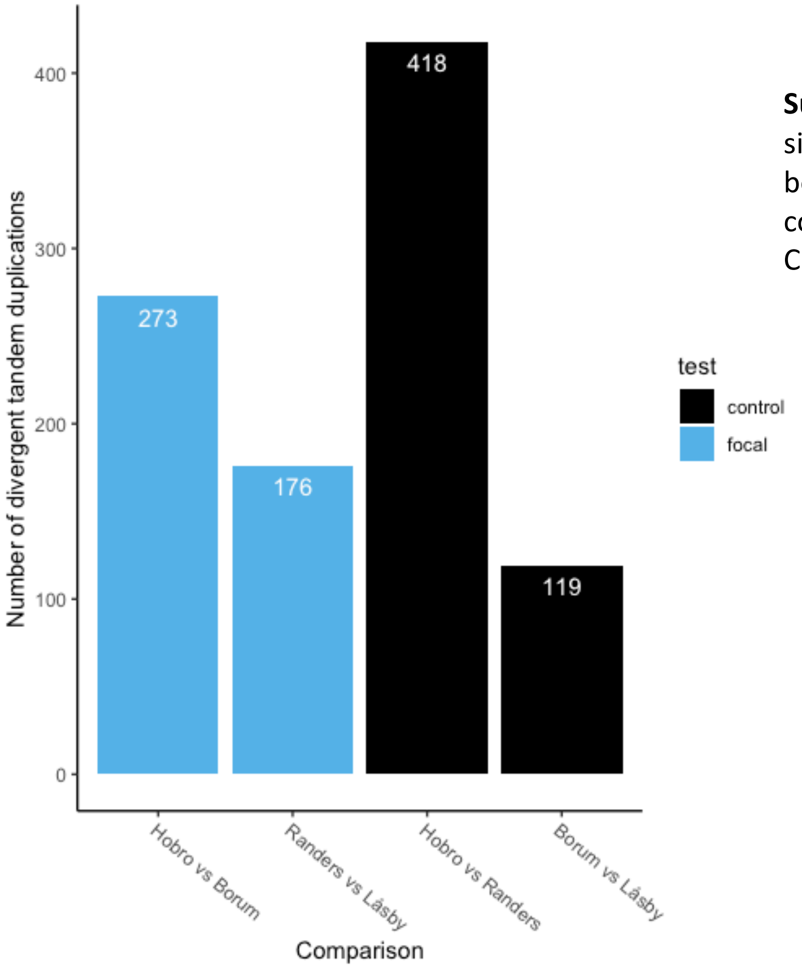

Supplementary Figure 5b Number of significantly divergent tandem duplications between each pairwise population comparison of the Focal Test and of the Control Test.

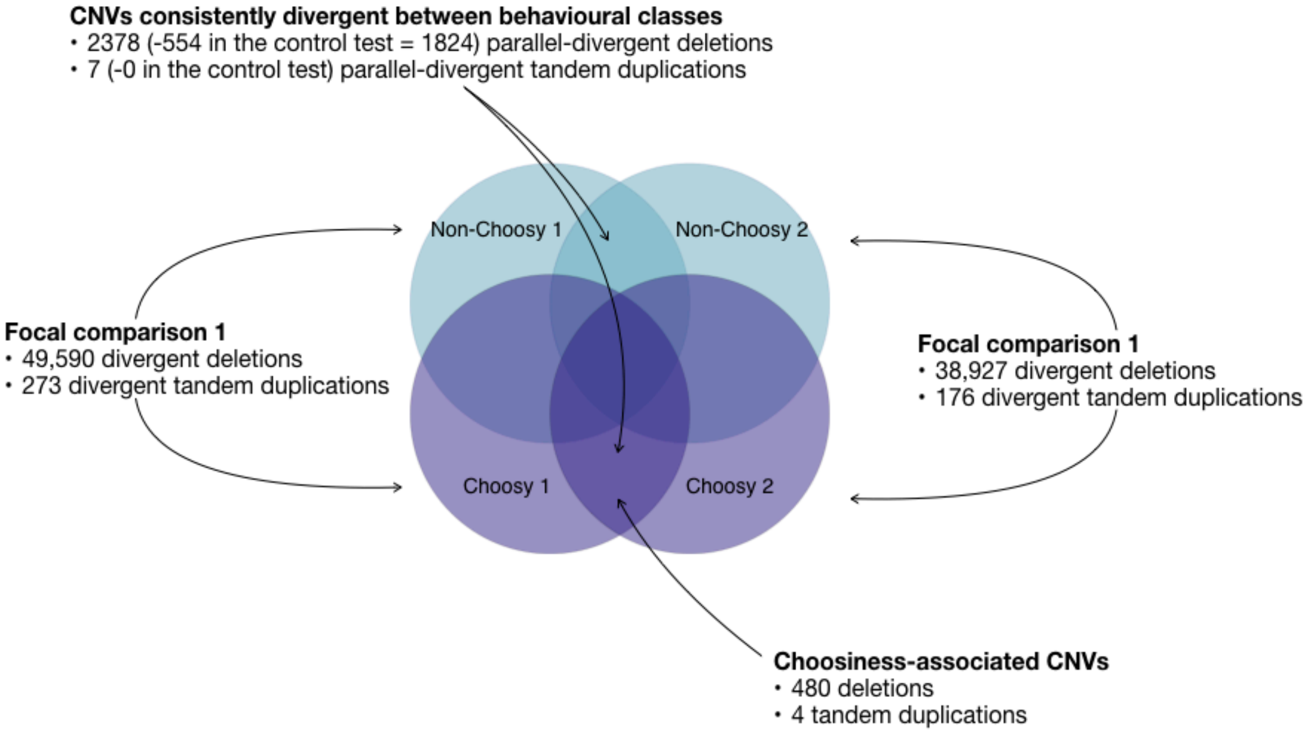

Supplementary Figure S6

Frequency of CNVs showing (1) divergence between pairs of Choosy-Non-Choosy populations in each comparison of the Focal Test, (2) consistent divergence between Choosy and Non-Choosy populations, and (3) copy-number displacement, i.e. choosiness-association.

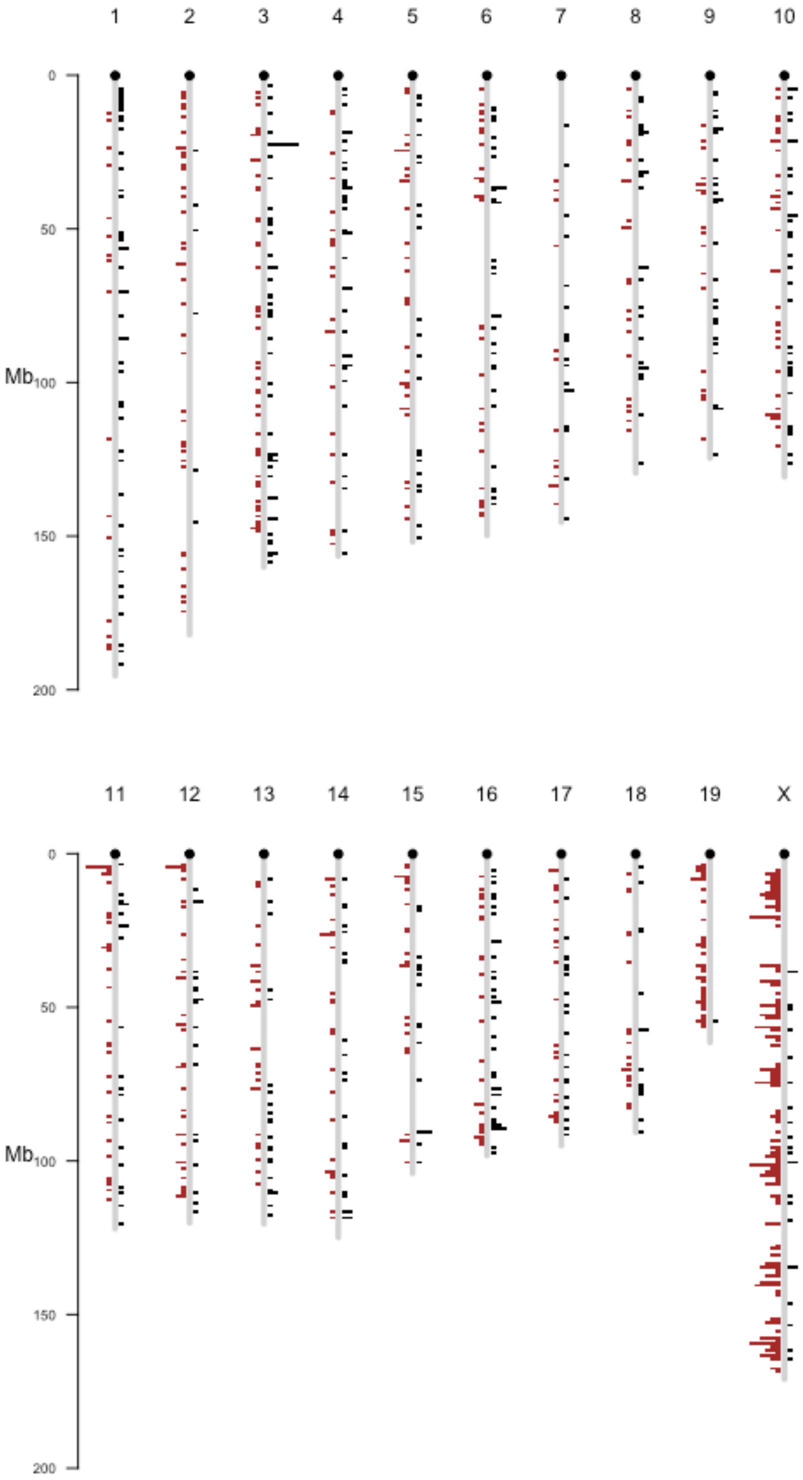

Supplementary Figure S7 Distribution of 484 Choosy-associated CNV compared to the distribution of recombination hotspots.

Black bar height (right) indicates the count of CNVs within each 10 kb genomic segment (minimum 1, maximum 5). Red bar height (left) indicates the count of recombination hotspots within each 10 kb genomic segment (minimum 1, maximum 5). Hotspots were identified as regions that were within the upper 95% percentile of ssDNA fragments per kilobase per million reported by Smagulova (2016).

Citations

1. Schrider DR, Begun DJ, Hahn MW. 2013 Detecting highly differentiated copy-number variants from pooled population sequencing. In *Biocomputing 2013*, pp. 344–355. World Scientific.

2. Schrider DR, Hahn MW, Begun DJ. 2016 Parallel evolution of copy-number variation across continents in Drosophila melanogaster. *Mol. Biol. Evol.* **33**, 1308–1316.

3. Cooper GM, Zerr T, Kidd JM, Eichler EE, Nickerson DA. 2008 Systematic assessment of copy number variant detection via genome-wide SNP genotyping. *Nat. Genet.* **40**, 1199.

4. Guan P, Sung W-K. 2016 Structural variation detection using next-generation sequencing data: a comparative technical review. *Methods* **102**, 36–49.
